# The Effect of Abiotic Factors on Abundance and Photosynthetic Performance of Airborne Cyanobacteria and Microalgae Isolated from the Southern Baltic Sea Region

**DOI:** 10.3390/cells10010103

**Published:** 2021-01-08

**Authors:** Kinga Wiśniewska, Sylwia Śliwińska-Wilczewska, Anita Lewandowska, Marta Konik

**Affiliations:** 1 Division of Marine Chemistry and Environmental Protection, Institute of Oceanography, University of Gdansk, Avenue Piłsudskiego 46, P-81-378 Gdynia, Poland; kinga.wisniewska@phdstud.ug.edu.pl (K.W.); anita.lewandowska@ug.edu.pl (A.L.); 2 Division of Marine Ecosystems Functioning, Institute of Oceanography, University of Gdansk, Avenue Piłsudskiego 46, P-81-378 Gdynia, Poland; 3 Department of Marine Physics, Institute of Oceanology Polish Academy of Sciences, P-81-779 Sopot, Poland; mk@iopan.gda.pl

**Keywords:** environmental stress, abiotic stressors, chlorophyll fluorescence, growth, photosynthetic pigments, plant physiology

## Abstract

Cyanobacteria and microalgae present in the aquatic or terrestrial environment may be emitted into the air and transported along with air masses over long distances. As a result of staying in the atmosphere, these organisms may develop a greater tolerance to stressful factors, but this topic is still relatively unknown. The main aim was to show an autecological characteristic of some airborne microalgae and cyanobacteria strains by a factorial laboratory experiment approach, including changes in irradiance, temperature, and salinity conditions. The additional purpose of this work was also to present part of the Culture Collection of Baltic Algae (CCBA) collection, which consists of airborne algae (AA) isolated from the atmospheric air of the southern Baltic Sea region. Altogether, 61 strains of airborne cyanobacteria and microalgae from the southern Baltic Sea region were isolated from May 2018 to August 2020. Selected microorganisms were tested in controlled laboratory conditions to identify their response to different irradiance (10–190 µmol photons m^−2^ s^−1^), temperature (13–23 °C), and salinity conditions (0–36 PSU). The highest numbers of cells (above 30 × 10^5^ cell mL^−1^) were recorded for cyanobacterium *Nostoc* sp., and for diatoms *Nitzschia* sp., *Amphora* sp., and *Halamphora* sp. We found that for cyanobacterium *Nostoc* sp. as well as for green alga *Coccomyxa* sp. the maximum cell concentrations were recorded at the salinity of 0 PSU. Moreover, cyanobacteria *Planktolyngbya contorta*, *Pseudanabaena catenata*, *Leptolyngbya foveolarum*, *Gloeocapsa* sp., and *Rivularia* sp. were able to grow only at a salinity of 0 PSU. On the other hand, in the range of 16–24 PSU, the highest cell numbers of examined diatoms have been identified. Our research provided that deposited airborne microalgae and cyanobacteria showed full colonization potential. The present experiment suggests that the adaptive abilities of microorganisms, in particular those producing toxins, may contribute to the spread in the future. Thus, it may increase human exposure to their negative health effects. Any distinctive adaptations of the genera give them an additional competitive advantage and a greater chance for territorial expansion.

## 1. Introduction

Cyanobacteria and microalgae present in the aquatic or terrestrial environment can be emitted into the air and transported along with air masses over long distances [1,2,3]. As of today, it is known that the cyanobacteria and microalgae present in the air play a role in processes occurring in the air and in global climate change [4,5]. Through absorbing and dispersing solar radiation, these species affect the radiation budget of the Earth and may also form ice-nucleating particles and cloud condensation nuclei [3,4,6,7]. There are also scientific reports that cyanobacteria and microalgae can, after the aerosolization process, invade the human respiratory tract and constitute a serious health risk [1,8,9,10,11]. Bioaerosols may be emitted from the water surface with the air bubbles and spray, which is correlated with the wind speed and wave conditions. The mechanisms of the emission of cyanobacteria and microalgae to the air are complex processes and were described in detail in several reviews [3,4,5]. Nevertheless, in both aerobiology and phycology, these microorganisms are the least understood [12,13].

The conditions in the atmosphere are certainly not favorable for the presence of cyanobacteria and microalgae. Bioaerosols, including cyanobacteria and microalgae present in the air, are exposed to stress factors such as temperature, humidity, oxidative stress, nitrogen starvation, radiation, and osmotic stress [2,3,4,5]. There is a theory suggesting that this stress is an evolutionary force that causes selection pressure and hence affects the spread and evolution of organisms [2]. The atmospheric survival capability depends on the adaptability of cyanobacteria and microalgae to changing environmental conditions [2,3,5]. The stress response is of great importance to their capability to colonize new habitats and to survive. Certain cyanobacteria can form sheath and mucilage, while microalgae form resistant stages [5,14]. They also tolerate demanding environmental conditions: irradiance, temperature, and humidity gradients [3,5,14,15]. According to Tesson et al. [5] and Jewson et al. [16], there is a possibility that microalgae may change the stage of life e.g., into dormant cells, during their stay in unfavorable weather conditions. Therefore, further investigation of the physiological modifications that affect airborne microalgae as they disperse is essential. Mathematical models exist to help understand the emission and transport of bioaerosols in the atmosphere, but do not provide information on the biological consequences for the organisms concerning survival, vitality, and metabolic activity [2]. Hence, the knowledge about these adaptations is still insufficient and requires environmental research and laboratory experiments.

The emergence of new phytoplankton species and changes in their taxonomical composition indicate changes in biodiversity in the Baltic Sea [17]. Ojaveer et al. [17] emphasize that the causes of biodiversity change are not yet fully understood, and the impact of biodiversity changes on the Baltic Sea ecosystem cannot be determined yet. The authors clarify that the possibility of introduction and acclimatization of new phytoplankton organisms to the Baltic Sea is enhanced by global patterns related to climate change [17]. Thus, the goal of our study was to indicate from which regions of the Baltic Sea the isolated airborne microalgae are originated. In addition, our research was aimed at demonstrating their potential for expansion range and the possibility of an invasion into other regions of the Baltic Sea and even further aquatic ecosystems. The study will extend the knowledge of algal biodiversity and help to preserve the microbial and biological resources of the region. Thus, cyanobacteria and microalgae strains from CCBA AA will help to investigate the biodiversity of the atmospheric environment, and through isolation, collection, and maintenance of new strains they can give information about their potentially negative impact on human health.

## 2. Results and Discussion

### 2.1. Characterization of Airborne Cyanobacteria and Microalgae Strains

**Figure 1:**
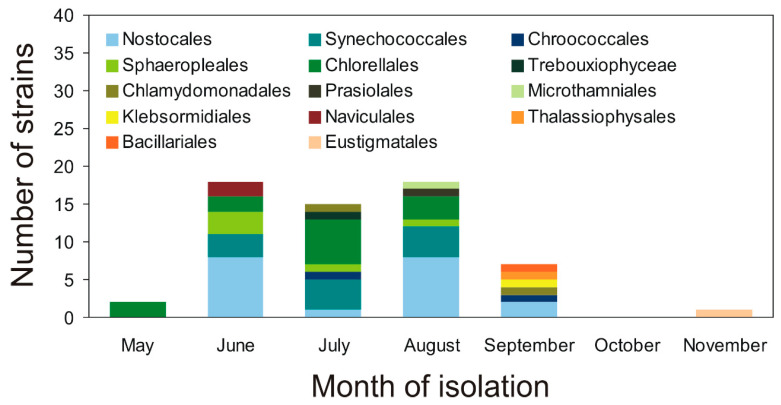
The number of airborne cyanobacteria and microalgae strains belonging to orders isolated in the study period (2018–2020).

Altogether, 61 strains of airborne cyanobacteria and microalgae belonging to 14 orders and 4 phyla from the southern Baltic Sea region were isolated from May 2018 to August 2020 (Figure 1). In scientific studies, the number of recorded organisms is given, but usually, they are not isolated and cultivated [3]. One of the largest amounts of cyanobacteria and microalgae in the air was recorded by Brown et al. [18] and it was 62 species. The success of the study was attributed to the diverse methods used by the authors to sample by using vehicle, aircraft, and normally-exposed Petri dishes at many sites. According to a review study conducted by Wiśniewska et al. [3], *Chlorella* sp. was the most common Chlorophyte in these bioaerosol studies, while *Chlorococcum* sp. was another that appeared regularly. Among the analyzed phylum, Cyanobacteria *Phormidium* sp. was the most common Cyanobacteria; however, *Lyngbya* sp., *Nostoc* sp. and *Anabaena* sp. were also frequently noted. On the other hand, *Navicula* sp. and *Nitzschia* sp. were the most commonly found Bacillariophyta [3]. A detailed analysis of the number of blue-green algae and microalgae identified in the air on a global scale was described by Wiśniewska et al. [3]. Analyzing the number of isolated strains, it was shown that cyanobacteria (Cyanophyta) occurred in June, July, August, and September 2018–2019 (Figure 1, Table S1). The highest number of cyanobacteria isolates were recorded in August (12 strains) and in June (11 strains). Among the isolated strains, cyanobacteria belonging to the orders Nostocales and Synechococcocales dominated in the study period. The green algae (Chlorophyta) were isolated from May 2018 to September 2020. The largest number of green algae strains were isolated in July (9 strains). The isolated green algae were also characterized by the highest diversity (6 orders). Green algae belonging to the orders Chlorellales and Sphaeropleales were the most numerous. In June and September 2018–2019, four diatom species (Bacillariophyta) belonging to 3 orders (Naviculales, Thalassiophysales, and Bacillariales) were also isolated. In addition, one strain belonging to Klebsormidiales (Charophyta) was isolated in September 2019, and one strain belonging to Eustigmatales (Ochrophyta) in November 2019. There have been months when individual strains could not be isolated, e.g., due to not enough organisms in the sample (Figure 1, Table S1).

As the largest number of isolated strains was recorded in the summer (June–August), it was decided to select three strains of cyanobacteria, green algae, and diatoms belonging to different orders for further research. In this study, we undertook the characterization of three strains of airborne cyanobacteria: *Nostoc* sp. (CCAA 03), *Synechococcus* sp. (CCAA 14), *Aphanothece* sp. (CCAA 48), green algae (B): *Oocystis* sp. (CCAA 20), *Coccomyxa* sp. (CCAA 21), *Kirchneriella* sp. (CCAA 38), and diatoms (C): *Nitzschia* sp. (CCAA 17), *Amphora* sp. (CCAA 34), *Halamphora* sp. (CCAA 47), which were the most abundant in aerosols of the Baltic Sea region. The listed organisms, depending on their preferences, can occur in various environments, from marine, through brackish, to freshwaters. However, the exact classification is often related to the species or strain of the microorganism [19].

**Figure 2:**
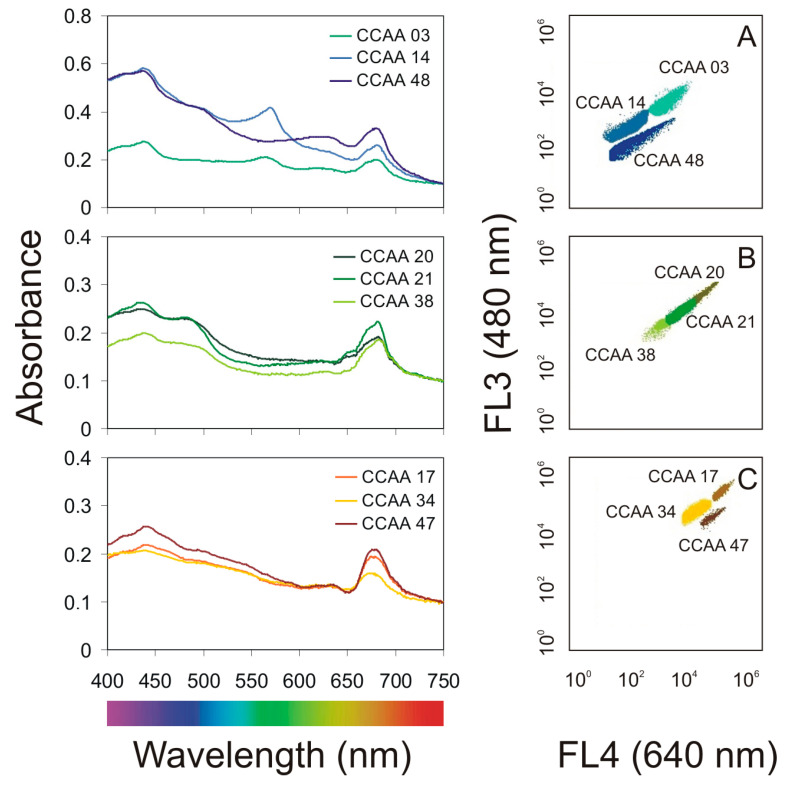
Left-hand panel—PAR absorbance spectra determined for the airborne cyanobacteria (**A**): *Nostoc* sp. (CCAA 03), *Synechococcus* sp. (CCAA 14), *Aphanothece* sp. (CCAA 48), green algae (**B**): *Oocystis* sp. (CCAA 20), *Coccomyxa* sp. (CCAA 21), *Kirchneriella* sp. (CCAA 38), and diatoms (**C**): *Nitzschia* sp. (CCAA 17), *Amphora* sp. (CCAA 34), *Halamphora* sp. (CCAA 47) at an optical density (OD_750_) = 0.1; right-hand panel—cytograms obtained using a BD Accuri C6 Plus flow cytometer. FL3 detectors (red fluorescence) read fluorescence emissions excited by the blue laser (480 nm), whereas the FL4 detector (orange fluorescence) reads emissions excited by the red laser (640 nm).

It was shown that with the same initial value (OD_750_ = 0.1), the tested strains differed in the absorbance spectrum (Figure 2). Apart from the visible peaks of chlorophyll *a* (Chl *a*), which have approximate absorbance maxima of 430 nm and 662 nm, phycobilin pigments (Phyco) are visible in cyanobacteria. Absorption peaks for phycoerythrin (PE) in the visible light spectrum are measured at 498 and 565 nm. Phycocyanin (PC) has a single absorption peak at 621 nm. In green algae, there is also a peak for chlorophyll *b* (Chl *b*), which has approximate maxima of 453 nm and 642 nm. While in diatoms there is a characteristic peak for chlorophyll *c* (Chl *c*), the absorption maxima of which are around 450, 581, and 629 nm. Cyanobacteria and microalgae have a variety of different pigments that can absorb energy from a wide range of wavelengths [20]. However, when an absorbance spectrum is performed on living organisms, the peaks may overlap, masking the view of the individual pigments. A more adequate way to characterize live strains is to perform a flow cytometer measurement. Thus, it can easily distinguish and identify selected airborne cyanobacteria and microalgae. It has been shown that depending on photosynthetic pigments, small organisms (*Synechococcus* sp. and *Aphanothece* sp.) are visible by the low signal intensity at 640 nm and 480 nm on the cytogram, while larger-sized organisms (*Nitzschia* sp., *Oocystis* sp.) and filamentous *Nostoc* sp. were marked by the high signal intensity at 640 nm and 480 nm on the cytogram (Figure 2). It is worth noticing that based on our observations, only the strains within the groups can be differentiated (although for green algae it would be difficult for accurate separation in a mixed sample). Some organisms from the same group (green algae, cyanobacteria, diatoms) with a different structure and cell size may not be ideal and separated.

Organisms obtained for the algae collections are usually isolated from seas and different inland waters [21,22]. This research focused on the isolation of airborne cyanobacteria and microalgae. At the moment, little data are allowing to determine the differences in the physiology of strains obtained from the air and the water samples [23]. However, it can be assumed that differences within one species may exist [21,24]. A great advantage of isolating cyanobacteria and microalgae from the atmosphere is the fact that it enables us to obtain species unusual for a given water reservoir or area because these organisms can be introduced from remote areas. The transport distance depends on the size and shape of particles. The large and heavy particles are dragging by the wind at a shorter distance. Small cells may be transported on hundreds of kilometers and then be deposited [1,4,5]. Studies are confirming the influence of meteorological parameters such as wind speed, air masses direction, air temperature, and humidity on the diversity of cyanobacteria and microalgae from the air [25,26]. However, the determination of their source of origin focuses on the use of the backward trajectories of air masses and the analysis of the taxonomic composition of potential sources location [3,26]. The isolation of species from the air can significantly enrich collection cultures of cyanobacteria and microalgae with organisms from distant regions. To the best of our knowledge, there is no significant algal culture collection maintaining airborne cyanobacteria and microalgae except for our newly established airborne algae (AA) collection, which is part of the Culture Collection of Baltic Algae (CCBA).

### 2.2. The Cell Concentration of Airborne Strains under Different Culture Conditions

The Baltic Sea is relatively shallow and small (the average depth of the Baltic Sea is 52.3 m, the maximum—459 m, and the area is approximately 415,266 km^2^) with limited water exchange through the narrow and shallow Danish Straits. Photosynthetically active radiation may fluctuate between 389 and 2117 µmol photons m^−2^ s^−1^ during the day [27]. Many different habitats and marine species are affected by pollution, fishing, physical modification, and other human activities, making the sea a harsh place for organisms to live [28,29]. The unique feature of the Baltic Sea is that there are areas where freshwater, brackish, and marine species occur [30]. Climatic diversity within the Baltic Sea shows the wide fluctuation in many physical and chemical parameters such as river runoff, salinity, sea level, and sea ice. Its salinity increases from east to west and north to south, from almost freshwater conditions in the northern Gulf of Bothnia, with salinity fluctuaing between 1–3 PSU, to almost oceanic conditions in the northern Kattegat, with salinity within the range 18–30 PSU. Sea surface temperature in the Proper Baltic varies from 1–2 °C in February up to 17–18 °C in August [30]. In such a variable ecosystem as the Baltic Sea region, autotrophic organisms should show the ability to adapt quickly, which is crucial for their survival and the possibility of settling in new areas. The future climate changes leading to global warming may favor the development of picocyanobacterial—the smallest cell-size cyanobacteria (0.2–2 µm) [31]—which under high temperatures achieve the maximum abundance [32,33]. Our research, conducted between 2018 and 2020 in the southern Baltic Sea region, showed that the increasing intensity of light and temperature had a positive effect on the cell concentration of the studied cyanobacterium *Nostoc* sp., and for diatoms *Nitzschia* sp., *Amphora* sp., and *Halamphora* sp.

**Figure 3:**
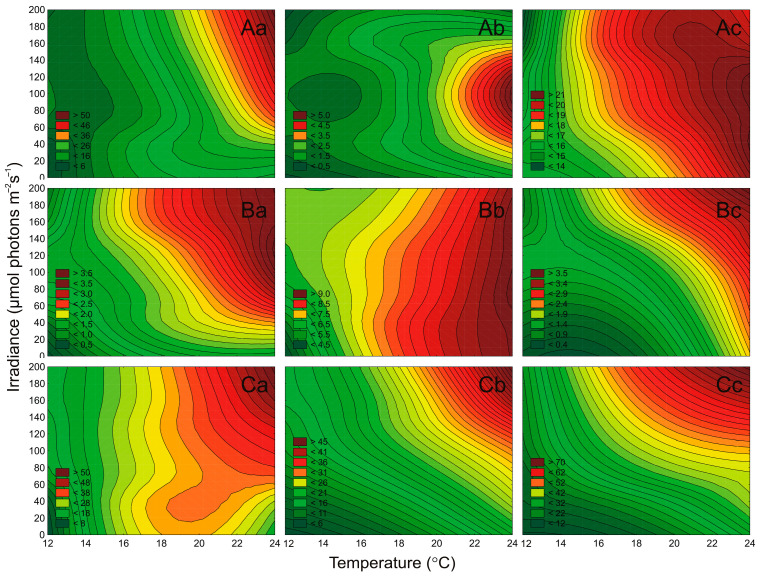
The number of cells (N × 10^5^ mL^−1^) obtained after 7 days of experiment for airborne cyanobacteria: *Nostoc* sp. (**Aa**), *Synechococcus* sp. (**Ab**), and *Aphanothece* sp. (**Ac**); airborne green algae: *Oocystis* sp. (**Ba**), *Coccomyxa* sp. (**Bb**), and *Kirchneriella* sp. (**Bc**); airborne diatoms: *Nitzschia* sp. (**Ca**), *Amphora* sp. (**Cb**), and *Halamphora* sp. (**Cc**) under different irradiance (μmol photons m^−2^ s^−1^) and temperature (°C) conditions.

In this study, the concentration of cyanobacteria and microalgae cells were determined under different irradiance and temperature conditions (Figure 3). In general, it was shown that both irradiance and temperature significantly affected the number of cells of examined airborne cyanobacteria (ANOVA, *p* < 0.001, *p* < 0.001, *p* < 0.01, for *Nostoc* sp., *Synechococcus* sp., and *Aphanothece* sp., respectively), green algae (ANOVA, *p* < 0.001, *p* < 0.05, *p* < 0.001, for *Oocystis* sp., *Coccomyxa* sp., and *Kirchneriella* sp., respectively), and diatoms (ANOVA, *p* < 0.001, *p* < 0.001, *p* < 0.001, for *Nitzschia* sp., *Amphora* sp., and *Halamphora* sp., respectively; Table S2). ANOVA results also showed that for each airborne strain, the effect of temperature on the cell concentration was higher than the influence of irradiance and the interaction of both factors simultaneously. It is worth mentioning here that irradiance had no significant effect on *Coccomyxa* sp. (ANOVA, *p* > 0.5; Table S2). *Coccomyxa* sp. is resistant to enduring particularly harsh environmental conditions, e.g., grown under a low and high temperature and even high irradiance [34]. Its low variability of cells number indicates a good adaptation of the harsh conditions during emissions from the water reservoir and transport in the atmosphere.

It was shown that for cyanobacterium *Nostoc* sp., green algae *Oocystis* sp. and *Kirchneriella* sp., and for all tested diatoms, the increase in light and temperature had a positive effect on the cell numbers in the cultures. In turn, cyanobacterium *Synechococcus* sp. showed growth inhibition in the highest tested irradiance. Moreover, picocyanobacteria *Synechococcus* sp. and *Aphanothece* sp. showed a significant increase in cell concentration with increasing temperature (Figure 3A). This indicates that in the future *Aphanothece* sp. and also *Synechococcus* sp. may become dominant in the Baltic Sea region and other aquatic ecosystems. Thus, if a new organism inhabits a given area, there is a high probability that if it acclimates there, it can show allelopathic effects on organisms already present there. The minimum number of cells was obtained for *Kirchneriella* sp. at 13 °C and 10 μmol photons m^−2^ s^−1^ (0.45 × 10^5^ cell mL^−1^; Figure 3B). The highest number of cells (71.66 × 10^5^ cell mL^−1^) was noted for *Halamphora* sp. at 190 μmol photons m^−2^ s^−1^ and 23 °C (Figure 3C). *Halamphora* sp., isolated from the Baltic Sea, has a wide temperature range of 15–25 °C, but a 30 °C-preferring strain has also been isolated. By higher temperatures, the authors noted a decrease in photosynthesis [35]. Moreover, high numbers of cells (above 30 × 10^5^ cell mL^−1^) were also recorded for diatoms *Nitzschia* sp. and *Amphora* sp. and for cyanobacterium *Nostoc* sp. (Figure 3) The latest research on airborne microalgae proves that these organisms have several adaptations, like better UV tolerance, that distinguish them from microalgae present in water [23]. Better adaptation of these organisms to stress treatment can give them the advantage to settle in new areas and become invasive species. It indicates that, after entering the appropriate environment, these species will easily attain substantial biomass and even become the dominant species in this aquatic ecosystem. It is worth noting here that *Nostoc* sp. is an important CyanoHABs species [36,37], as well as *Nitzschia* sp. as a Red Tide organism [38]. The presence of microorganisms that produce toxins in the air, and their ability to spread and acclimate to new environmental conditions is of particular importance in terms of its impact on human health [3].

**Figure 4:**
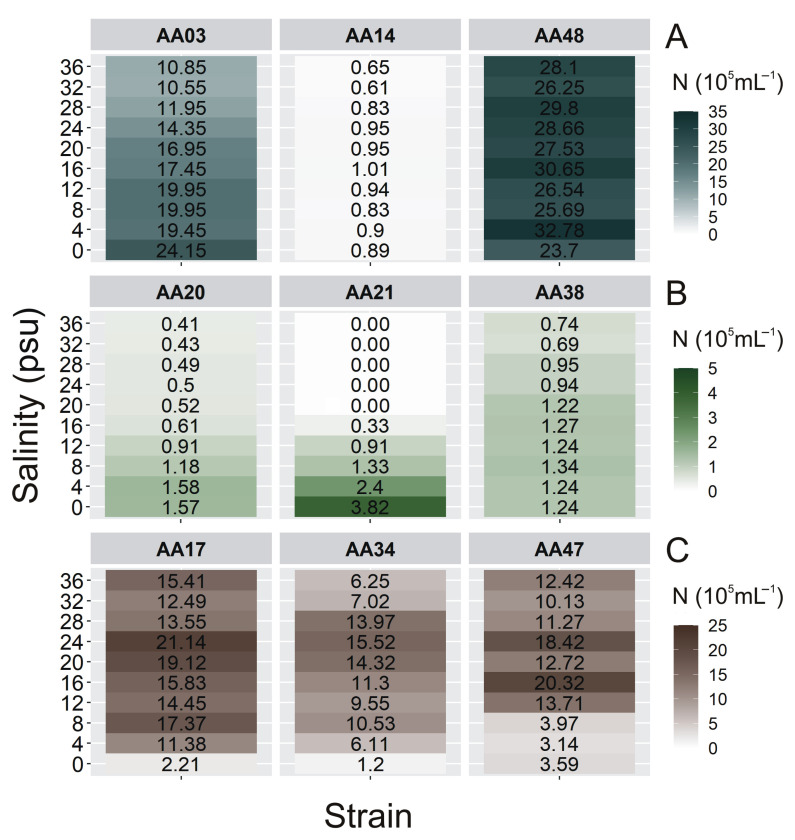
The number of cells (N × 10^5^ mL^−1^) obtained after 7 days of experiment for airborne cyanobacteria (**A**): *Nostoc* sp. (CCAA 03), *Synechococcus* sp. (CCAA 14), *Aphanothece* sp. (CCAA 48), green algae (**B**): *Oocystis* sp. (CCAA 20), *Coccomyxa* sp. (CCAA 21), *Kirchneriella* sp. (CCAA 38), and diatoms (**C**): *Nitzschia* sp. (CCAA 17), *Amphora* sp. (CCAA 34), *Halamphora* sp. (CCAA 47) under different salinity conditions.

Assessing the origin of microorganisms that are present in the air in small amounts is a difficult task. It is often based solely on the analysis of the backward air masses trajectory. Hence it is worth enriching them with laboratory experiments such as the analysis of the number of organisms under different salinity conditions. To estimate the region of origin of airborne cyanobacteria and microalgae, additional experiments have been performed to determine the effect of salinity on the cells’ number (Figure 4). In our work, we used different salinity of culture medium to isolate new strains of airborne cyanobacteria and microalgae. Based on our observations, we have shown that most of the CCAA strains grow satisfactorily at the salinity medium of 8 PSU. However, there are several strains (*Planktolyngbya contorta* CCAA11, *Pseudanabaena catenata* CCAA12, *Leptolyngbya foveolarum* CCAA15, *Gloeocapsa* sp. CCAA18, *Pseudanabaena catenata* CCAA19, and *Rivularia* sp. CCAA 49) that grow at 0 PSU salinity.

In general, one-way ANOVA tests showed that salinity significantly affected the number of cells of examined airborne cyanobacteria (*p* < 0.001, for all), green algae (*p* < 0.001, for all), and diatom (*p* < 0.001, for all; Table S3). It was found that for cyanobacterium *Nostoc* sp. (Figure 4A) as well as for green alga *Coccomyxa* sp. (Figure 4B) the maximum cell concentration was recorded at the salinity of 0 PSU (24.15 × 10^5^ cell mL^−1^ and 3.82 × 10^5^ cell mL^−1^, respectively). It is worth mentioning here that *Coccomyxa* sp. did not grow at salinity above 16 PSU, despite numerous information related to their wide tolerance to harsh environmental conditions including salinity [34]. Moreover, the highest cell numbers of tested diatoms were found in the range of 16–24 PSU (21.14 × 10^5^ cell mL^−1^ for *Nitzschia* sp., 15.52 × 10^5^ cell mL^−1^ for *Amphora* sp., and 20.32 × 10^5^ cell mL^−1^ for *Halamphora* sp.; Figure 4C).

Our measurements have shown that salinity has also a significant impact on the concentration of cells of individual species of algae and cyanobacteria. It was found that for cyanobacterium *Nostoc* sp. as well as for green alga *Coccomyxa* sp. the maximum cell concentrations were recorded at the salinity of 0 PSU (Figure 4A,B). Moreover, cyanobacteria *Planktolyngbya contorta*, *Pseudanabaena catenata*, *Leptolyngbya foveolarum*, *Gloeocapsa* sp., and *Rivularia* sp. belonging to the CCAA were able to grow only at a salinity of 0 PSU (data not shown). It suggests that these species come from freshwater communities. Research conducted by Woelfel et al. [27] indicates that salinity did not strongly influence the growth of diatoms, they also have a high tolerance to low salinity. In contrast, diatoms isolated from the air had the highest cell numbers found in the range of 16–24 PSU; however, all of them had lower tolerance to 0 PSU (Figure 4C). According to Potapova [39], the disparity in salt content between coastal and inland waters was considered a significant barrier that only a few diatom lineages were able to cross in one direction, from marine to freshwater habitat. These findings indicate that the airborne diatoms may come from the western part of the Baltic Sea [17]. Surprisingly, it was found that the picocyanobacteria *Synechococcus* sp. and *Aphanothece* sp. showed a similar abundance in the whole range of tested salinity. Considering their small cell size and their ability to be transported with aerosols over long distances, this property may give them an advantage in colonizing new water areas even with various salinity. The HYSPLIT air mass trajectories model [40,41] let us determine whether the given cyanobacteria and microalgae present in the air were brought from near or far, land or sea source areas [1,3]. We noted that only in the case of *Nitzschia* sp. and *Aphanothece* sp. Did the analysis of the backward trajectories of air masses indicated their transport from over the land (Figure S1e). When analyzing the salinity preferences of selected strains, with the highest abundance by 4 PSU, it can be also concluded that *Aphanothece* sp. had a terrestrial origin. The preference for *Aphanothece* sp. to freshwater is confirmed by literature data [42]. In addition, Dembowska [43] showed the presence of *Aphanothece* sp. in the waters of lakes (Iławskie Lake District) over which the mass of air passed through.

In all other cases, the analysis of taxa origin was much more difficult because the air masses passed through the Southern Baltic Proper first and then through the land. In the case of *Synechococcus* sp., *Oocystis* sp., *Coccomyxa* sp., and *Halamphora* sp., the air masses flowed over the measuring station all the way from the North Sea (Figure S1b). Analyzing salinity preferences in combination with the backward trajectories of air masses, it is most likely that only *Halamphora* sp. could have arrived from the distant Kattegat region (Figure S1a).

### 2.3. Effect of Irradiance and Temperature on Pigments Content

**Figure 5:**
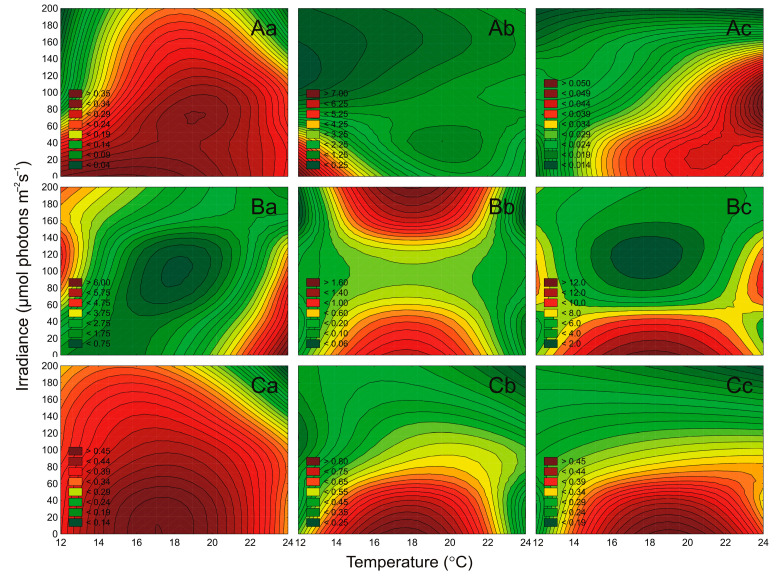
Changes in Chl *a* content (pg cell^−1^) obtained after 7 days of experiment for airborne cyanobacteria: *Nostoc* sp. (Aa), *Synechococcus* sp. (**Ab**), and *Aphanothece* sp. (**Ac**); airborne green algae: *Oocystis* sp. (**Ba**), *Coccomyxa* sp. (**Bb**), and *Kirchneriella* sp. (**Bc**); airborne diatoms: *Nitzschia* sp. (**Ca**), *Amphora* sp. (**Cb**), and *Halamphora* sp. (**Cc**) under different irradiance (μmol photons m^−2^ s^−1^) and temperature (°C) conditions.

**Figure 6:**
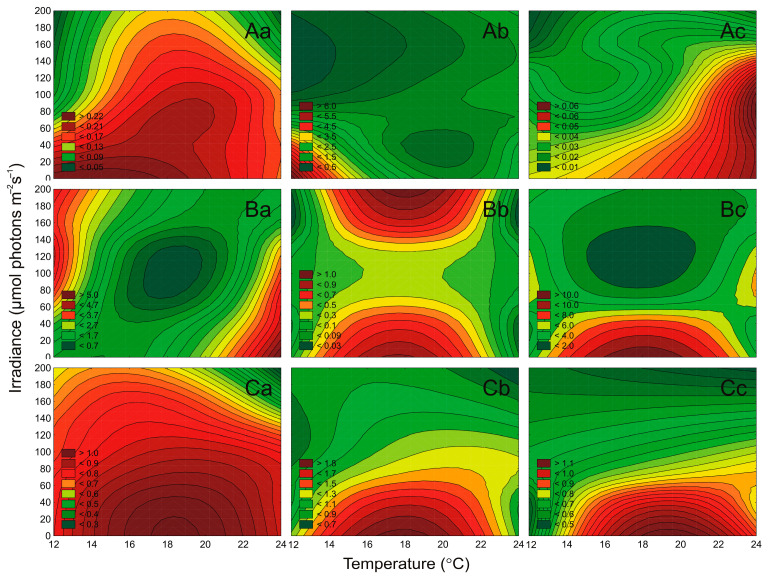
Changes in Car content (pg cell^−1^) obtained after 7 days of experiment for airborne cyanobacteria: *Nostoc* sp. (**Aa**), *Synechococcus* sp. (**Ab**), and *Aphanothece* sp. (**Ac**); airborne green algae: *Oocystis* sp. (**Ba**), *Coccomyxa* sp. (**Bb**), and *Kirchneriella* sp. (**Bc**); airborne diatoms: *Nitzschia* sp. (**Ca**), *Amphora* sp. (**Cb**), and *Halamphora* sp. (**Cc**) under different irradiance (μmol photons m^−2^ s^−1^) and temperature (°C) conditions.

The autecology of airborne cyanobacteria and microalgae was characterized by the changes in composition and proportion of photosynthetic pigments, i.e., Chl *a*, Chl *b*, Chl *c*, and Phyco under different irradiance (μmol photons m^−2^ s^−1^) and temperature (°C) conditions. As Chl *a* is the main photosynthetic pigment, and Car is responsible for protecting the microorganisms and is a variable when cells are under stress, they are presented in Figure 5 and Figure 6. While additional pigments (Phyco, Chl *b*, Chl *c*) were presented in the Supplementary Materials Figure S2.

It was found that irradiance and temperature as well as their interaction significantly affected the Chl *a* content in *Nostoc* sp., *Synechococcus* sp., and *Aphanothece* sp. (ANOVA, *p* < 0.001, *p* < 0.001, and *p* < 0.001, respectively), as well as Car content (ANOVA, *p* < 0.001, *p* < 0.001, and *p* < 0.001, respectively). ANOVA indicated that for examined cyanobacteria, the effect of irradiance on cell-specific pigments content was higher than the effect of temperature and the interaction of both factors (Table S4). It was shown that the cell-specific pigments content (Chl *a*, Car) for *Nostoc* sp. and *Synechococcus* sp. decreased with increasing irradiance (Figure 5 and Figure 6). On the other hand, for *Aphanothece* sp. a decrease in Chl *a* (Figure 5(Ac)) and Car content (Figure 6(Ac)) with increasing irradiance was shown. The highest content of the studied pigments was found in *Synechococcus* sp., while the lowest was in *Aphanothece* sp. The highest content of Chl *a* (Figure 5(Ab)) and Car (Figure 6(Ab)) was recorded in *Synechococcus* sp. At the lowest irradiance (10 μmol photons m^−2^ s^−1^) and the lowest temperature (13 °C), which is respectively 5.77 pg cell^−1^ and 4.69 pg cell^−1^.

Irradiance and temperature, as well as their interaction, were found to significantly affect the Chl *a* content in tested strains of green algae (ANOVA, *p* < 0.001, for *Oocystis* sp., *p* < 0.001, for *Coccomyxa* sp., and *p* < 0.001, for *Kirchneriella* sp.), and Car content (ANOVA, *p* < 0.001, for all). For Chl *a*, and Car content, the effect of temperature for *Oocystis* sp. And *Coccomyxa* sp. Was higher than the effect of irradiance and the interaction of both factors (Table S5). In turn, the Chl *a* and Car of *Kirchneriella* sp. Was more affected by irradiance than by temperature. It was shown that the cell-specific pigments content (Chla *a* and Car) for the analyzed green algae decreased with increasing irradiance at 13 °C and 23 °C. On the other hand, at the temperature of 18 °C, the content of the analyzed pigments remained at a similar level. The lowest Chla *a*, and Car content were recorded for *Coccomyxa* sp. On the other hand, it was demonstrated that *Kirchneriella* sp. Had the highest concentration of cell-specific pigments content was compared to examined green algae. The highest content of Chla *a* (Figure 5(Bc)) and Car (Figure 6(Bc)) was recorded at irradiance 10 μmol photons m^−2^ s^−1^ and temperature 18 °C, which were 12.84 pg cell^−1^ and 10.33 pg cell^−1^ respectively.

Both irradiance and temperature significantly affected also the cell-specific Chl *a* content of *Nitzschia* sp., *Amphora* sp., and *Halamphora* sp. (ANOVA, *p* < 0.001, for all), as well as Car content (ANOVA, *p* < 0.001, for all). ANOVA indicated that the effect of irradiance on pigments content was higher than the effect of temperature and of the relationship of both factors (Table S6). It was shown that the cell-specific pigments content for the analyzed diatoms decreased with increasing irradiance (Figure 5C and Figure 6C). In general, the lowest cell-specific pigment content for the analyzed diatoms was recorded at 190 μmol photons m^−2^ s^−1^ and 23 °C. The highest content of Chla *a* and Car was recorded for *Amphora* sp., which in the light of 10 μmol photons m^−2^ s^−1^ and temperature of 18 °C was equal to 0.85 pg cell^−1^ and 1.90 pg cell^−1^ respectively. Moreover, it was also shown that *Amphora* sp. had the highest concentration of cell-specific pigment content compared to other diatoms.

For many cyanobacteria and microalgae, the high light intensity is an unfavorable environmental factor [44], which organisms can respond to by, for example, changing the concentration of pigments in cells [23,32,45]. Generally, the factorial experiments performed in this study showed a negative effect of the increasing irradiance on the content of the cell-specific pigment for the analyzed airborne cyanobacteria and microalgae, obtaining the highest contents at 10 μmol photons m^−2^ s^−1^ and the lowest for 190 μmol photons m^−2^ s^−1^. It was shown that only the cell-specific Phyco content for *Aphanothece* sp. increased with increasing light (Figure S2). High Chl *a* content in low light may indicate that airborne cyanobacteria and microalgae may deposit in highly shaded waters and continue to grow intensively [46]. The increased chlorophyll content found in microalgae grown at low light intensity is possibly due to light/shaded adaptations that enhance the use of light energy [47]. It is also worth noting that in the cases of cyanobacteria and diatoms, irradiance has a stronger effect on pigments than temperature.

Moreover, under low irradiance, the greatest increase of Phyco in cyanobacterial cells was noted (Figure S2), which additionally confirms the ability of these species to appear in shaded waters [48,49]. It can be because photoaclimatization occurs when photosynthetic pigments are reduced with the increase of irradiation intensity [50]. Thus, in the case of isolated in the region of the southern Baltic Sea airborne cyanobacteria and microalgae, except *Aphanothece* sp., the photoaclimatization ability was noted, which proves the ability to adapt to changing environmental conditions. It is also worth mentioning here that stressful factors such as high light intensity favor the accumulation of Car pigments that have a protective function. The latest research on airborne green-algae has shown that these organisms accumulate high levels of carotenoids in such stressful conditions [23]. However, in the present study, this phenomenon was not observed, which may suggest that the isolated microorganisms adapted to these conditions and were not under stress. According to the “Everything small is everywhere” hypothesis, not much can be done to prevent airborne microalgae and cyanobacteria to colonize new regions, when the environmental factors change. Thus, such experiments are especially important for estimating future effects in a changing environment.

### 2.4. Effect of Irradiance and Temperature on the Maximum PSII Quantum Efficiency

The effect of irradiance and temperature on changes in the maximum quantum efficiency of PSII photochemistry (*F*_v_/*F*_m_ parameter) of airborne cyanobacteria and microalgae strains was also determined. It was found that irradiance and temperature, as well as their interaction significantly affected the *F*_v_/*F*_m_ of tested cyanobacteria (ANOVA, *p* < 0.001, for all), green algae (ANOVA, *p* < 0.001, for all), and diatoms (ANOVA, *p* < 0.001, for *Amphora* sp., and *Halamphora* sp., respectively). It was also shown that irradiance and temperature had no significant effect on *Nitzschia* sp. (ANOVA, *p* > 0.05; Table S7). Generally, ANOVA indicated that for tested airborne cyanobacteria, green algae, and diatoms the effect of temperature on the *F*_v_/*F*_m_ parameter was higher than the effect of irradiance and the interaction of both factors (Table S7).

**Figure 7:**
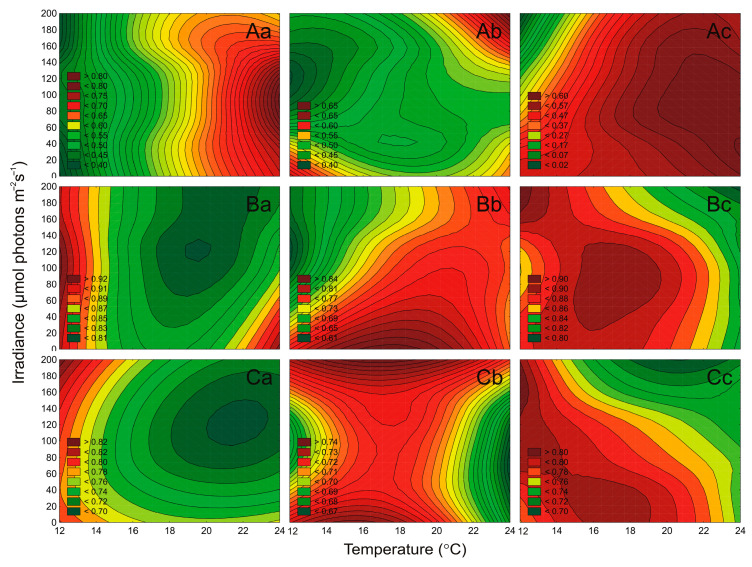
Changes in the maximum PSII quantum efficiency (*F*_v_/*F*_m_) obtained after 7 days of experiment for airborne cyanobacteria: *Nostoc* sp. (**Aa**), *Synechococcus* sp. (**Ab**), and *Aphanothece* sp. (**Ac**); airborne green algae: *Oocystis* sp. (**Ba**), *Coccomyxa* sp. (**Bb**), and *Kirchneriella* sp. (**Bc**); airborne diatoms: *Nitzschia* sp. (**Ca**), *Amphora* sp. (**Cb**), and *Halamphora* sp. (**Cc**) under different irradiance (μmol photons m^−2^ s^−1^) and temperature (°C) conditions.

**Figure 8:**
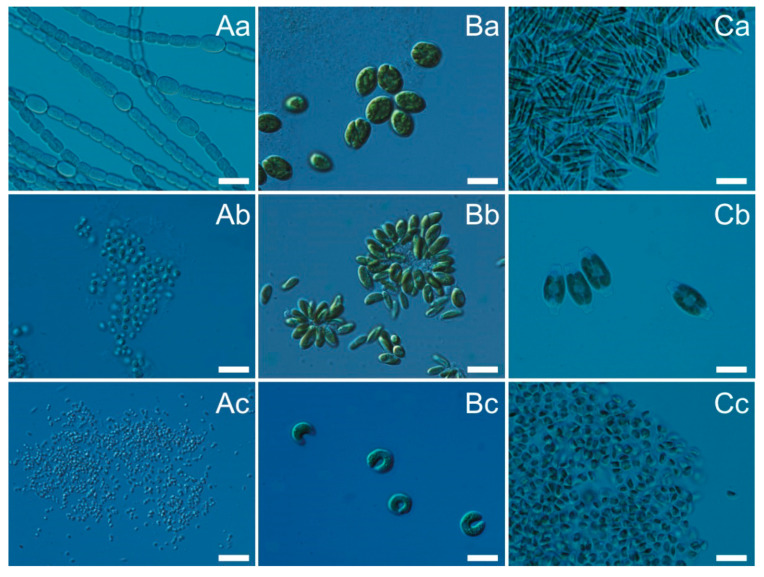
Photographs of airborne cyanobacteria (**A**): *Nostoc* sp. (CCAA 03; (**a**)), *Synechococcus* sp. (CCAA 14; (**b**)), *Aphanothece* sp. (CCAA 48; (**c**)), green algae (**B**): *Oocystis* sp. (CCAA 20; (**a**)), *Coccomyxa* sp. (CCAA 21; (**b**)), *Kirchneriella* sp. (CCAA 38; (**c**)), and diatoms (**C**): *Nitzschia* sp. (CCAA 17; (**a**)), *Amphora* sp. (CCAA 34; (**b**)), *Halamphora* sp. (CCAA 47; (**c**)) used in this study. Scale bar = 10 μm.

It was shown that for the tested cyanobacteria, an increase in the *F*_v_/*F*_m_ value with increasing temperature was noted. In turn, a decrease in *F*_v_/*F*_m_ with increasing irradiance was also recorded. The highest values of *F*_v_/*F*_m_ among the examined cyanobacteria, as in the case of the number of cells, were recorded for *Nostoc* sp. The highest value of the Fv/Fm parameter, equal to 0.76, for this cyanobacterium, occurred under 100 μmol photons m^−2^ s^−1^ and 23 °C (Figure 7A). In general, for green algae and diatoms, a decrease in *F*_v_/*F*_m_ was recorded with increasing irradiance (Figure 7B,C). The greatest differences were recorded for *Coccomyxa* sp. at 13 °C. In such conditions, the decrease of this parameter from 10 to 190 μmol photons m^−2^ s^−1^ was more than 1.2-fold (Figure 7B). Moreover, green algae were characterized by the highest values of the *F*_v_/*F*_m_ parameter compared to cyanobacteria and diatoms. For *Oocystis* sp. and *Kirchneriella* sp. the *F*_v_/*F*_m_ always exceeded the value of 0.8 (Figure 7B). In turn, the lowest *F*_v_/*F*_m_ value (0.12) among all the analyzed strains was recorded for *Aphanothece* sp. at irradiance 190 μmol photons m^−2^ s^−1^ and temperature 13 °C (Figure 8A).

The chlorophyll *a* fluorescence measurements may indicate changes in photosystem II (PSII) activity by calculating the maximum quantum yield of PSII (*F*_v_/*F*_m_) [51]. On the basis of our research, we determined that the studied airborne cyanobacteria and microalgae showed differences in the functioning of the maximum quantum yield of PSII, depending on the environmental factors. In general, for cyanobacteria, green algae, and diatoms, a decrease in the maximum quantum yield of PSII was recorded with increasing light. It was shown that for the tested cyanobacteria, an increase in the maximum quantum yield of PSII with increasing temperature was noted. The green algae were characterized by the highest values of the *F*_v_/*F*_m_ parameter compared to cyanobacteria and diatoms. In turn, the lowest *F*_v_/*F*_m_ value among all the analyzed strains was recorded for *Aphanothece* sp. The appropriate composition of photosynthetic pigments, which is responsible for the proper functioning of the photosynthesis mechanism, enables airborne cyanobacteria and microalgae to grow under the control of changing environmental conditions and to colonize different water bodies [20].

## 3. Materials and Methods

### 3.1. Culture Conditions

All airborne cyanobacteria and microalgae were collected between 2018 and 2020 at a research station in Gdynia, located 1 km from the coastal zone of the Gulf of Gdańsk, southern Baltic Sea region [1]. Algae have been identified by were identified using keys and world literature [52,53,54,55,56,57,58]. The taxonomic composition was analyzed under a Nikon Eclipse 80i microscope at a magnification of 10 and 100×. A list of species isolated in the study period is shown in Table S1 (in Supplementary Material). The factorial experiments were conducted on airborne cyanobacterial strains: *Nostoc* sp. (CCAA 03), *Synechococcus* sp. (CCAA 14), *Aphanothece* sp. (CCAA 48), green algae strains: *Oocystis* sp. (CCAA 20), *Coccomyxa* sp. (CCAA 21), *Kirchneriella* sp. (CCAA 38), and diatoms strains: *Nitzschia* sp. (CCAA 17), *Amphora* sp. (CCAA 34), *Halamphora* sp. (CCAA 47) (Figure 8).

The strains isolated from the air over the Baltic Sea region are maintained as unialgal cultures in the Culture Collection of Baltic Algae (Airborne Algae—AA) at the Institute of Oceanography, University of Gdańsk, Poland. Tests on the “batch cultures” were carried out in 25-mL glass Erlenmeyer flasks containing sterilized F/2 medium [59]. The strains were incubated under a 16:8 h light:dark cycle at three PAR irradiances (10, 100, and 190 μmol photons m^−2^ s^−1^), at three temperatures (13 °C, 18 °C, and 23 °C), and salinity of 8 PSU. For the experiment determining the abundance of the tested strains under the influence of different salinity, ten different F/2 mediums (in the range of 0 to 36 PSU) were made. These mediums were obtained by dissolving the appropriate amount of Tropic Marin Sea Salt in a specific volume of distilled water. The salinity of the medium was measured by a salinometer (inoLabCond Level 1, Weilheim in Oberbayern, Germany).

The initial number of cells in all conducted experiments was 10^5^ cells per mL. The test cultures were grown in three replicates and were incubated for one week. After that time in the exponential growth phase, the cells concentration, pigments content, as well as photosynthesis performance, were measured in each replicate.

### 3.2. Calculation of Cell Density

Cell density was calculated using linear regression models based on cell concentration (N mL^−1^) and optical density (OD). Calculation of the cell number was conducted using the procedure described by Śliwińska-Wilczewska et al. [60] with a BD Accuri C6 Plus flow cytometer (BD Biosciences, San Jose, CA, USA) or by Śliwińska-Wilczewska et al. [61] with a light microscope (Nikon Eclipse 80i, Nikon, Tokyo, Japan) and the Bürker counting chamber for filamentous cyanobacteria. This method makes it possible to determine the correlation coefficients and the linear correlations between the number of cells and OD. The cell concentrations in the test cultures were estimated on the basis of calibration curves (Table S8).

### 3.3. Air Masses Trajectories

For each sampling day, using 6-h intervals, the air mass 48 h backward trajectory was obtained using the HYSPLIT model (NOAA Air Resources Laboratory, College Park, MD, USA) [40,41]. HYSPLIT is a complete system for computing basic trajectories of air parcels for dynamic simulations of dispersion and deposition. The three standard air mass (500, 1000, and 1500 m AGL) arrival heights were used to test the location of air mass overlap. By tracking the path of air masses at a given time 48h before sampling, this information helps to determine the source of both chemical and biological air pollution.

### 3.4. Determination of the Chlorophyll, Carotenoids, and Phycobiliproteins Content

The concentration of chlorophyll (Chl *a*) for tested cyanobacterial and microalgal strains was calculated according to Jeffrey and Humphrey [62]. The concentration of carotenoids (Car) was calculated using the formula employed by Strickland and Parsons [63]. After 7 days of incubation, 20 mL of culture was filtered using 0.45 µm filters (Macherey-Nagel MN GF-5, Dueren, Germany). Chl *a* and Car were extracted with cold 90% acetone in the dark for 2 h at −20 °C. To remove cell debris and filter particles the pigment extract was centrifuged at 12,000× *g* rpm for 2 min (Sigma 2-16P, Osterode am Harz, Germany). The extinction was determined at 750 nm, 665 nm, and 480 nm with a UV-VIS Multiskan GO spectrophotometer (Thermo Scientific, Waltham, MA, USA) and using 1 cm glass cuvette.

Phycobiliproteins (Phyco) were extracted for tested cyanobacterial strains according to Stewart and Farmer [64]. After 7 days of incubation, 20 mL of culture was filtered using 0.45 µm filters (Macherey-Nagel MN GF-5, Dueren, Germany). Each filter was thoroughly homogenized in a medium consisting of 0.25 M Trizma Base, 10 mM disodium EDTA, and 2 mg/mL lysozyme. The pH of the medium was adjusted to 5.0 through the addition of hydrochloric acid (concentrated HCl). Homogenates were incubated in darkness for 2 h at 37 °C and then for 20 h at 2 °C. To remove cell debris and filter particles, the pigment extract was centrifuged at 12,000× *g* rpm for 2 min (Sigma 2-16P, Osterode am Harz, Germany). The absorbance of the pigment extract was measured at 565 nm, 620 nm, 650 nm, and 750 nm with a Multiskan GO spectrophotometer (Thermo Scientific, Waltham, MA, USA) and using a 1 cm glass cuvette. The concentration of Phyco was calculated according to Tandeau de Marsac and Houmard [65].

### 3.5. Determination of the Chlorophyll a Fluorescence

The measurement of the chlorophyll fluorescence of airborne cyanobacteria and microalgae was conducted after 7 days of the experiment according to the method described by Śliwińska-Wilczewska et al. [24]. Fluorescence parameter *F*_v_/*F*_m_ (where *F*_v_—the difference between the maximum and minimum fluorescence and *F*_m_—the maximum fluorescence) [51] was analyzed using pulse amplitude modulated (PAM) fluorometer (FMS1, Hansatech, King’s Lynn, United Kingdom). Illumination was provided by a 594 nm amber modulating beam with 4 step frequency control. Cyanobacterial and microalgal materials were placed in the leaf clip on the 13 mm glass fiber filter (Whatman GF/C, Saint Louis, MO, USA). The actinic light was the same as the one used for the growth of algal cells to provide optimal conditions for photosynthetic activity. Saturation pulses (4500 μmol photons m^−2^ s^−1^) of 0.7 s duration were used for all airborne cyanobacterial and microalgal species. All samples were dark-adapted for 5 min before measurements.

### 3.6. Statistical Analyses

To test the influence of a light intensity, temperature, and their interaction on studied parameters the two-way ANOVA was used. One-way ANOVA was used to test the effect of salinity on the number of cells of tested strains on the last day of the experiment. Levels of significance were: * *p* < 0.05; ** *p* < 0.01; *** *p* < 0.001. All data are reported as means ± standard deviations (SD). The statistical analyses were performed using Statistica^®^ 13.1 software (TIBCO Software Inc., StatSoft, Kraków, Poland). The heat maps were performed using RStudio 1.3.1056 software (Boston, MA, USA).

## 4. Conclusions

Transport of bioaerosols with air masses may play important role in microbial dispersal, and thus maintaining the diversity of water systems [5,66]. To date, the invasiveness of new species has been closely connected to their transfer from the basin to the basin along with the ballast waters. Almost all known harmful algal bloom species have been reported in viable form from this source [67]. However, nowadays research on airborne algae confirms that these organisms can be carried far distances from their source of origin [1,5,68], thus it is worth testing their preferences and backward trajectories in order to estimate their source. Our research has shown that cyanobacteria *Nostoc* sp. as well as green alga *Coccomyxa* sp. may come from freshwater communities. On the other hand, the highest cell numbers of analyzed diatoms were found in the range of 16–24 PSU. These findings indicate that the airborne diatoms may come from the western part of the Baltic Sea. Moreover, analyzing salinity preferences in combination with the backward trajectories of air masses, it is most likely that only *Halamphora* sp. could have arrived from the distant Kattegat region. It was found that the picocyanobacteria *Synechococcus* sp. and *Aphanothece* sp. showed a similar abundance in the whole range of tested salinity. Considering picocyanobacterial small cell size and their ability to be transported with aerosols over long distances, this property may give them an advantage in colonizing new water areas even with various salinity conditions. We also found that cyanobacterium *Nostoc* sp., and diatom *Nitzschia* sp., a well-known bloom-forming species, can quickly reach significant biomass and even become the dominant species in the aquatic ecosystem as they showed the highest numbers of cells under favorable light, temperature, and salinity conditions. The present experiment suggests that the adaptive abilities of microorganisms, in particular those producing toxins, may contribute to the spread in the future. Thus, it may increase human exposure to their negative health effects.

Airborne cyanobacteria and microalgae still remain a group of poorly understood organisms due to difficulties in their extraction and isolation. Moreover, the increase in the number of alien and invasive species is becoming an increasingly serious problem in the world. Therefore, it is necessary to carefully examine the physiology and autecology of airborne organisms and their ability to inhabit distant water bodies. Isolating and creating a collection of cyanobacteria and microalgae has great potential. These organisms can later be used in environmental studies, education, or biotechnology [21]. In addition, the isolation of individual strains allows their use in laboratory conditions exploring the relationships between organisms present in the natural environment [21]. Thanks to this, the complex relationships that occur in nature can be better understood.

## Data Availability

Data is contained within the article or supplementary material.

## Supplementary Materials

The following are available online at https://www.mdpi.com/2073-4409/10/1/103/s1. Figure S1: 48-h air mass backward trajectory analysis from HYSPLIT model (Draxler and Hess, 1998, NOAA Air Resources Laboratory, US) for the date of sampling; Figure S2: Changes in Phyco (A; pg·cell^−1^), Chl *b* (B; pg·cell^−1^), and Chl *c* (C; pg·cell^−1^) content obtained after 7 days of experiment for airborne cyanobacteria; Table S1: A list of species from the southern Baltic Sea region isolated in 2018-2020.; Table S2: Two-way factorial ANOVA of cells concentration measured in tested airborne cyanobacteria and microalgae growing at different temperatures (°C) and irradiance (μmol photons m^−2^ s^−1^); Table S3: One-way ANOVA of cells concentration measured in tested airborne cyanobacteria and microalgae growing at different salinities (PSU); Table S4: Two-way factorial ANOVA of cell-specific Chl *a*. Car. and Phyco content measured in tested airborne cyanobacteria growing at different temperatures (°C) and irradiance (μmol photons m^−2^ s^−1^); Table S5: Two-way factorial ANOVA of cell-specific Chl *a*. Car. and Chl *b* content measured in tested airborne green algae growing at different temperatures (°C) and irradiance (μmol photons m^−2^ s^−1^); Table S6: Two-way factorial ANOVA of cell-specific Chl *a*. Car. and Chl *c* content measured in tested airborne diatoms growing at different temperatures (°C) and irradiance (μmol photons m^−2^ s^−1^); Table S7: Two-way factorial ANOVA of *F*_v_/*F*_m_ parameter measured in tested airborne cyanobacteria and microalgae growing at different temperatures (°C) and irradiance (μmol photons m^−2^ s^−1^); Table S8: Linear regression and correlation coefficients (r) used to calculate the number (*N*) of studied airborne cyanobacteria, green algae, and diatoms cells in cultures based on optical density (OD) measurements.
