# Supplementary material for: The Effect of Abiotic Factors on Abundance and Photosynthetic Performance of Airborne Cyanobacteria and Microalgae Isolated from the Southern Baltic Sea Region
Source: Cells. 2021 Jan 8;10(1):103. doi: 10.3390/cells10010103 (PMC7826845; doi:10.3390/cells10010103)
Supplement: Supplementary file 1 [file cells-10-00103-s001.pdf]

Supplementary material

Table S1. A list of species from the southern Baltic Sea region isolated in 2018-2020.

Number	Species	Order	Phylum	Date of isolation
CCAA 23	*Chlorella* sp.	Chlorellales	Chlorophyta	22.05.18
CCAA 24	*Chlorella minutissima*	Chlorellales	Chlorophyta	24.05.18
CCAA 47	*Halamphora* sp.	Naviculales	Bacillariophyta	05.06.18
CCAA 49	*Rivularia* sp.	Nostocales	Cyanobacteria	22.06.18
CCAA 10	*Chlorella minutissima*	Chlorellales	Chlorophyta	24.06.18
**CCAA 38**	*Kirchneriella* sp.	Sphaeropleales	Chlorophyta	24.06.18
CCAA 39	*Nostoc edaphicum*	Nostocales	Cyanobacteria	24.06.18
CCAA 40	*Nostoc edaphicum*	Nostocales	Cyanobacteria	24.06.18
CCAA 26	*Pseudanabaena catenata*	Synechococcales	Cyanobacteria	24.06.18
CCAA 25	*Scenedesmus* sp.	Sphaeropleales	Chlorophyta	24.06.18
CCAA 44	*Synechococcus* sp.	Synechococcales	Cyanobacteria	24.06.18
CCAA 43	*Bracteacoccus* sp.	Sphaeropleales	Chlorophyta	26.06.18
CCAA 27	*Chlorella* sp.	Chlorellales	Chlorophyta	26.06.18
CCAA 41	*Navicula* sp.	Naviculales	Bacillariophyta	26.06.18
CCAA 42	*Nostoc edaphicum*	Nostocales	Cyanobacteria	26.06.18
CCAA 13	*Pseudanabaena catenata*	Synechococcales	Cyanobacteria	26.06.18
CCAA 30	*Nostoc* sp.	Nostocales	Cyanobacteria	29.06.18
CCAA 31	*Nostoc* sp.	Nostocales	Cyanobacteria	29.06.18
CCAA 32	*Nostoc* sp.	Nostocales	Cyanobacteria	29.06.18
CCAA 33	*Nostoc* sp.	Nostocales	Cyanobacteria	29.06.18
**CCAA 21**	*Cocomyxa* sp.	Trebouxiophyceae	Chlorophyta	06.07.18
**CCAA 20**	*Oocystis* sp.	Chlorellales	Chlorophyta	06.07.18
CCAA 9	*Oocystis* sp.	Chlorellales	Chlorophyta	06.07.18
**CCAA 14**	*Synechococcus* sp.	Synechococcales	Cyanobacteria	06.07.18
CCAA 18	*Gloeocapsa* sp.	Chroococcales	Cyanobacteria	08.07.18
CCAA 11	*Planktolyngbya contorta*	Synechococcales	Cyanobacteria	08.07.18
CCAA 12	*Pseudanabaena catenata*	Synechococcales	Cyanobacteria	08.07.18
CCAA 7	*Oocystis* sp.	Chlorellales	Chlorophyta	22.07.18
CCAA 8	*Pseudanabaena galeata*	Synechococcales	Cyanobacteria	22.07.18
CCAA 28	*Chlorella* sp.	Chlorellales	Chlorophyta	26.07.18
CCAA 29	*Nostoc* sp.	Nostocales	Cyanobacteria	26.07.18
CCAA 6	*Kirchneriella* sp.	Sphaeropleales	Chlorophyta	08.08.18
CCAA 15	*Leptolyngbya foveolarum*	Synechococcales	Cyanobacteria	08.08.18
CCAA 35	*Nostoc edaphicum*	Nostocales	Cyanobacteria	08.08.18
CCAA 36	*Nostoc edaphicum*	Nostocales	Cyanobacteria	08.08.18
CCAA 37	*Nostoc edaphicum*	Nostocales	Cyanobacteria	08.08.18
CCAA 1	*Nostoc* sp.	Nostocales	Cyanobacteria	08.08.18
CCAA 2	*Nostoc* sp.	Nostocales	Cyanobacteria	08.08.18
**CCAA 3**	*Nostoc* sp.	Nostocales	Cyanobacteria	08.08.18
CCAA 5	*Nostoc* sp.	Nostocales	Cyanobacteria	08.08.18
CCAA 19	*Pseudanabaena catenata*	Synechococcales	Cyanobacteria	08.08.18
CCAA 46	*Synechococcus* sp.	Synechococcales	Cyanobacteria	08.08.18
CCAA 4	*Nostoc* sp.	Nostocales	Cyanobacteria	12.08.18
CCAA 45	*Synechococcus* sp.	Synechococcales	Cyanobacteria	12.08.18
**CCAA 34**	*Amphora* sp.	**Thalassiophysales**	**Bacillariophyta**	**03.09.18**
**CCAA 48**	*Aphanothece* sp.	Chroococcales	Cyanobacteria	15.09.18
**CCAA 17**	*Nitzschia* sp.	Bacillariales	Bacillariophyta	15.09.18
CCAA 16	*Nostoc* sp.	Nostocales	Cyanobacteria	15.09.18
CCAA 22	*Nostoc* sp.	Nostocales	Cyanobacteria	15.09.18
CCAA 50	*Bracteacoccus* sp.	Sphaeropleales	Chlorophyta	11.07.19
CCAA 52	*Chlorella minutissima*	Chlorellales	Chlorophyta	15.07.19
CCAA 51	*Chlorococcum* sp.	Chlamydomonadales	Chlorophyta	15.07.19
CCAA 53	*Chlorella minutissima*	Chlorellales	Chlorophyta	30.07.19
CCAA 54	*Oocystis* sp.	Chlorellales	Chlorophyta	20.08.19
CCAA 55	*Oocystis* sp.	Chlorellales	Chlorophyta	20.08.19

CCAA 56	*Stichococcus* sp.	Prasiolales	Chlorophyta	22.08.19
CCAA 57	*Microthamnion* sp.	Microthamniales	Chlorophyta	29.08.19
CCAA 59	*Chlorococcum* sp.	Chlamydomonadales	Chlorophyta	11.09.19
CCAA 60	*Klebsormidium* sp.	Klebsormidiales	Charophyta	26.09.19
CCAA 61	*Vaucheria* sp.	Eustigmatales	Ochrophyta	15.11.19
CCAA 58	*Pseudococcomyxa* sp.	Chlorellales	Chlorophyta	04.08.20

Table S2. Two-way factorial ANOVA of cells concentration measured in tested airborne cyanobacteria and microalgae growing at different temperatures (°C) and irradiance (µmol photons $m^{-2}s^{-1}$): df – degrees of freedom; F – Fisher's F-test statistic; Mss – mean sum of squares; Ss – sum of squares. Levels of significance were: * $p < 0.05$; ** $p < 0.01$; *** $p < 0.001$.

Airborne strain	Source of variation	Df	Ss	Mss	F
Nostoc sp.	temperature	2	2967.207	1483.603	2203.4***
	irradiation	2	657.887	328.943	488.5***
	interaction	4	750.493	187.623	278.6***
	error	18	12.120	0.673	
Synechococcus sp.	temperature	2	19.024	9.512	2996.8***
	irradiation	2	7.063	3.531	1112.6***
	interaction	4	12.557	3.139	989.1***
	error	18	0.057	0.003	
Aphanothece sp.	temperature	2	100.332	50.166	131.3***
	irradiation	2	4.237	2.118	5.5*
	interaction	4	10.123	2.531	6.6**
	error	18	6.878	0.382	
Oocystis sp.	temperature	2	11.316	5.658	8674.0***
	irradiation	2	5.598	2.799	4290.7***
	interaction	4	1.734	0.433	664.4***
	error	18	0.012	0.001	
Coccomyxa sp.	temperature	2	43.276	21.638	68.3***
	irradiation	2	0.721	0.360	1.1
	interaction	4	4.059	1.015	3.2*
	error	18	5.703	0.317	
Kirchneriella sp.	temperature	2	13.753	6.877	4765.2***
	irradiation	2	7.832	3.916	2713.6***
	interaction	4	1.558	0.389	269.9***
	error	18	0.026	0.001	
Nitzschia sp.	temperature	2	2080.396	1040.198	221.6***
	irradiation	2	406.978	203.489	43.4***
	interaction	4	453.866	113.466	24.2***
	error	18	84.485	4.694	
Amphora sp.	temperature	2	1339.544	669.772	1678.8***
	irradiation	2	1150.185	575.092	1441.5***
	interaction	4	185.780	46.445	116.4***
	error	18	7.181	0.399	
Halamphora sp.	temperature	2	3526.776	1763.388	1301.2***
	irradiation	2	4270.001	2135.000	1575.5***
	interaction	4	678.096	169.524	125.1***
	error	18	24.393	1.355	

Table S3. One-way ANOVA of cells concentration measured in tested airborne cyanobacteria and microalgae growing at different salinities (PSU): df – degrees of freedom; F – Fisher's F-test statistic; Mss – mean sum of squares; Ss – sum of squares. Levels of significance were: * $p < 0.05$; ** $p < 0.01$; *** $p < 0.001$.

Airborne strain	Source of variation	Df	Ss	Mss	F
Nostoc sp.	factor	9	554.247	61.583	150.9***
	error	20	8.160	0.408	
Synechococcus sp.	factor	9	0.475	0.053	110.8***
	error	20	0.010	0.000	
Aphanothece sp.	factor	9	188.303	20.923	18.9***
	error	20	22.204	1.110	
Oocystis sp.	factor	9	5.867	0.652	2220.6***
	error	20	0.006	0.000	
Coccomyxa sp.	factor	9	45.942	5.105	70.6***
	error	20	1.447	0.072	
Kirchneriella sp.	factor	9	1.504	0.167	87.3***
	error	20	0.038	0.002	
Nitzschia sp.	factor	9	724.888	80.543	80.1***
	error	20	20.109	1.005	
Amphora sp.	factor	9	542.343	60.260	55.2***
	error	20	21.839	1.092	
Halamphora sp.	factor	9	963.536	107.060	19.4***
	error	20	110.236	5.512	

Table S4. Two-way factorial ANOVA of cell-specific Chl *a*. Car. and Phyco content measured in tested airborne cyanobacteria growing at different temperatures (°C) and irradiance (μmol photons $m^{-2}s^{-1}$): df – degrees of freedom; F – Fisher's F-test statistic; Mss – mean sum of squares; Ss – sum of squares. Levels of significance were: * $p < 0.05$; ** $p < 0.01$; *** $p < 0.001$.

Airborne strain	Cell-specific pigment content	Source of variation	Df	Ss	Mss	F
Nostoc sp.	Chl *a*	temperature	2	0.047	0.023	83.6***
		irradiation	2	0.121	0.061	216.6***
		interaction	4	0.033	0.008	29.2***
		error	18	0.005	0.000	
	Car	temperature	2	0.013	0.006	53.8***
		irradiation	2	0.040	0.020	166.4***
		interaction	4	0.013	0.003	26.3***
		error	18	0.002	0.000	
	Phyco	temperature	2	2.002	1.001	12.2***
		irradiation	2	8.274	4.137	50.3***
		interaction	4	5.915	1.479	18.0***
		error	18	1.481	0.082	
Synechococcus sp.	Chl *a*	temperature	2	4.092	2.046	479.1***
		irradiation	2	28.779	14.389	3370.2***
		interaction	4	30.246	7.562	1771.0***
		error	18	0.077	0,004	
	Car	temperature	2	3.118	1.559	555.3***
		irradiation	2	14.986	7.493	2669.3***
		interaction	4	19.857	4.964	1768.4***
		error	18	0.051	0.003	
	Phyco	temperature	2	58.298	29.149	37.8***
		irradiation	2	384.537	192.269	249.5***
		interaction	4	48.500	12.125	15.7***
		error	18	13.870	0.771	
Aphanothece sp.	Chl *a*	temperature	2	0.001	0.000	54.6***
		irradiation	2	0.002	0.001	89.7***
		interaction	4	0.001	0.000	17.5***
		error	18	0.000	0.000	
	Car	temperature	2	0.001	0.000	250.0***
		irradiation	2	0.001	0.000	394.2***
		interaction	4	0.000	0.000	60.1***
		error	18	0.000	0.000	
	Phyco	temperature	2	1.028	0.514	99.6***
		irradiation	2	1.894	0.947	183.4***
		interaction	4	1.639	0.410	79.4***
		error	18	0.093	0.005	

Table S5. Two-way factorial ANOVA of cell-specific Chl *a*. Car. and Chl *b* content measured in tested airborne green algae growing at different temperatures (°C) and irradiance (μmol photons $m^{-2}s^{-1}$): df – degrees of freedom; F – Fisher's F-test statistic; Mss – mean sum of squares; Ss – sum of squares. Levels of significance were: * $p < 0.05$; ** $p < 0.01$; *** $p < 0.001$.

Airborne strain	Cell-specific pigment content	Source of variation	Df	Ss	Mss	F
Oocystis sp.	Chl *a*	temperature	2	13.820	6.910	1844.9***
		irradiation	2	0.840	0.420	112.2***
		interaction	4	33.655	8.414	2246.3***
		error	18	0.067	0.004	
	Car	temperature	2	11.904	5.952	1685.2***
		irradiation	2	0.636	0.318	90.0***
		interaction	4	21.610	5.403	1529.6***
		error	18	0.064	0.004	
	Chl *b*	temperature	2	2.516	1.258	1304.1***
		irradiation	2	0.120	0.060	62.4***
		interaction	4	2.219	0.555	575.1***
		error	18	0.017	0.001	
Coccomyxa sp.	Chl *a*	temperature	2	4.184	2.092	2251.0***
		irradiation	2	0.863	0.432	464.5***
		interaction	4	1.840	0.460	494.9***
		error	18	0.017	0.001	
	Car	temperature	2	2.005	1.003	1996.9***
		irradiation	2	0.422	0.211	420.0***
		interaction	4	0.781	0.195	389.1***
		error	18	0.009	0.001	
	Chl *b*	temperature	2	0.302	0.151	458.4***
		irradiation	2	0.063	0.031	95.3***
		interaction	4	0.102	0.025	77.3***
		error	18	0.006	0.000	
Kirchneriella sp.	Chl *a*	temperature	2	1.007	0.504	6.5**
		irradiation	2	137.506	68.753	892.6***
		interaction	4	117.333	29.333	380.8***
		error	18	1.386	0.077	
	Car	temperature	2	0.413	0.206	4.3*
		irradiation	2	88.913	44.457	916.7***
		interaction	4	72.483	18.121	373.6***
		error	18	0.873	0.048	
	Chl *b*	temperature	2	1.022	0.511	49.3***
		irradiation	2	14.399	7.200	694.4***
		interaction	4	12.441	3.110	300.0***
		error	18	0.187	0.010	

Table S6. Two-way factorial ANOVA of cell-specific Chl *a*. Car. and Chl *c* content measured in tested airborne diatoms growing at different temperatures (°C) and irradiance (μmol photons $m^{-2}s^{-1}$): df – degrees of freedom; F – Fisher's F-test statistic; Mss – mean sum of squares; Ss – sum of squares. Levels of significance were: * $p < 0.05$; ** $p < 0.01$; *** $p < 0.001$.

Airborne strain	Cell-specific pigment content	Source of variation	Df	Ss	Mss	F
Nitzschia sp.	Chl *a*	temperature	2	0.048	0.024	142.9***
		irradiation	2	0.077	0.039	230.1***
		interaction	4	0.013	0.003	19.0***
		error	18	0.003	0.000	
	Car	temperature	2	0.076	0.038	36.0***
		irradiation	2	0.469	0.235	223.8***
		interaction	4	0.096	0.024	22.9***
		error	18	0.019	0.001	
	Chl *c*	temperature	2	0.005	0.002	221.4***
		irradiation	2	0.009	0.004	392.0***
		interaction	4	0.004	0.001	87.0***
		error	18	0.000	0.000	
Amphora sp.	Chl *a*	temperature	2	0.135	0.067	108.1***
		irradiation	2	0.378	0.189	303.0***
		interaction	4	0.137	0.034	54.8***
		error	18	0.011	0.001	
	Car	temperature	2	0.496	0.248	69.2***
		irradiation	2	2.146	1.073	299.5***
		interaction	4	0.570	0.142	39.8***
		error	18	0.064	0.004	
	Chl *c*	temperature	2	0.009	0.004	157.5***
		irradiation	2	0.020	0.010	356.5***
		interaction	4	0.005	0.001	42.5***
		error	18	0.001	0.000	
Halamphora sp.	Chl *a*	temperature	2	0.013	0.007	74.8***
		irradiation	2	0.123	0.061	685.7***
		interaction	4	0.027	0.007	74.2***
		error	18	0.002	0.000	
	Car	temperature	2	0.103	0.052	116.3***
		irradiation	2	0.606	0.303	684.8***
		interaction	4	0.174	0.044	98.4***
		error	18	0.008	0.000	
	Chl *c*	temperature	2	0.005	0.003	122.9***
		irradiation	2	0.006	0.003	136.6***
		interaction	4	0.002	0.001	27.4***
		error	18	0.000	0.000	

Table S7. Two-way factorial ANOVA of F_v/F_m parameter measured in tested airborne cyanobacteria and microalgae growing at different temperatures (°C) and irradiance (μmol photons $m^{-2}s^{-1}$): df – degrees of freedom; F – Fisher's F-test statistic; Mss – mean sum of squares; Ss – sum of squares. Levels of significance were: * $p < 0.05$; ** $p < 0.01$; *** $p < 0.001$.

Airborne strain	Source of variation	Df	Ss	Mss	F
Nostoc sp.	temperature	2	0.284	0.142	768.2***
	irradiation	2	0.004	0.002	9.9**
	interaction	4	0.025	0.006	33.7***
	error	18	0.003	0.000	
Synechococcus sp.	temperature	2	0.027	0.013	17.5***
	irradiation	2	0.020	0.010	13.4***
	interaction	4	0.039	0.010	12.8***
	error	18	0.014	0.001	
Aphanothece sp.	temperature	2	0.370	0.185	481.7***
	irradiation	2	0.112	0.056	146.1***
	interaction	4	0.120	0.030	78.1***
	error	18	0.007	0.000	
Oocystis sp.	temperature	2	0.023	0.011	1184.7***
	irradiation	2	0.003	0.002	180.2***
	interaction	4	0.006	0.001	151.9***
	error	18	0.000	0.000	
Coccomyxa sp.	temperature	2	0.038	0.019	117.0***
	irradiation	2	0.038	0.019	117.5***
	interaction	4	0.023	0.006	36.0***
	error	18	0.003	0.000	
Kirchneriella sp.	temperature	2	0.013	0.006	713.0***
	irradiation	2	0.002	0.001	106.3***
	interaction	4	0.006	0.002	171.8***
	error	18	0.000	0.000	
Nitzschia sp.	temperature	2	0.022	0.011	26.4***
	irradiation	2	0.005	0.002	5.7*
	interaction	4	0.004	0.001	2.3
	error	18	0.007	0.000	
Amphora sp.	temperature	2	0.004	0.002	690.4***
	irradiation	2	0.003	0.002	537.9***
	interaction	4	0.001	0.000	76.0***
	error	18	0.000	0.000	
Halamphora sp.	temperature	2	0.013	0.006	803.4***
	irradiation	2	0.010	0.005	601.0***
	interaction	4	0.007	0.002	224.8***
	error	18	0.000	0.000	

Table S8. Linear regression and correlation coefficients (r) used to calculate the number (*N*) of studied airborne cyanobacteria, green algae, and diatoms cells in cultures based on optical density (OD) measurements.

Studied strain	Linear regression	Correlation coefficient (r)
Nostoc sp.	N = 30000000·OD + 95000	0.97
Synechococcus sp.	N = 1743239·OD + 10699	0.99
Aphanothece sp.	N = 42545088·OD − 69136	0.99
Oocystis sp.	N = 1076436·OD − 11399	0.92
Coccomyxa sp.	N = 24898588·OD − 274132	0.96
Kirchneriella sp.	N = 1307250·OD − 11969	0.95
Nitzschia sp.	N = 15907560·OD − 44587	0.98
Amphora sp.	N = 21054196·OD − 13493	0.98
Halamphora sp.	N = 22813892·OD + 100824	0.98

where N—cells in 1 mL of medium and OD—optical density of the culture.

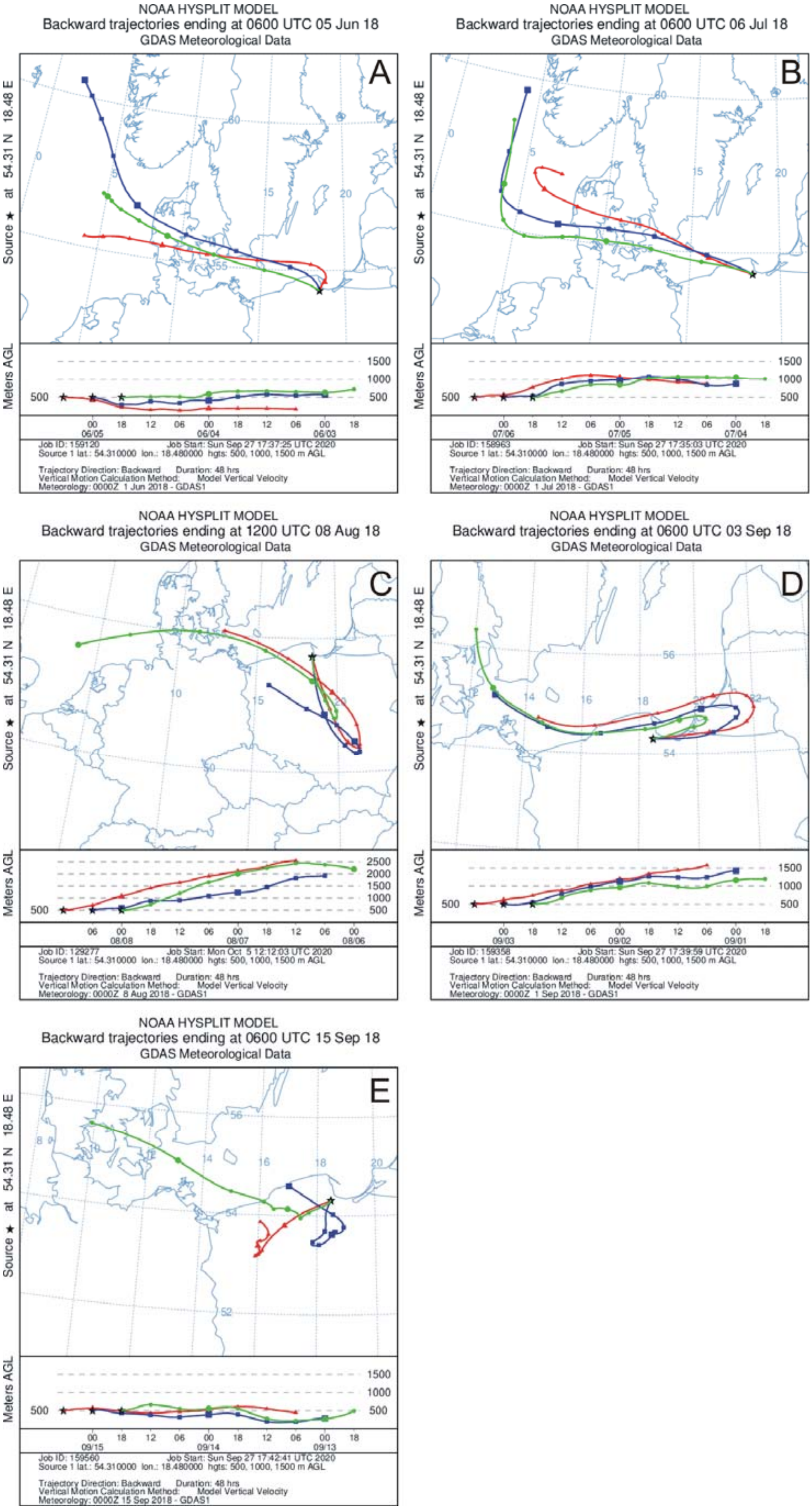

Figure S1. 48-h air mass backward trajectory analysis from HYSPLIT model (Draxler and Hess, 1998, NOAA Air Resources Laboratory, US) for the date of sampling: **A**) *Halamphora* sp., **B**) *Synechococcus* sp., *Oocystis* sp., *Cocomyxa* sp., **C**) *Nostoc* sp., **D**) *Amphora* sp., E) *Nitzschia* sp. and *Aphanothece* sp.

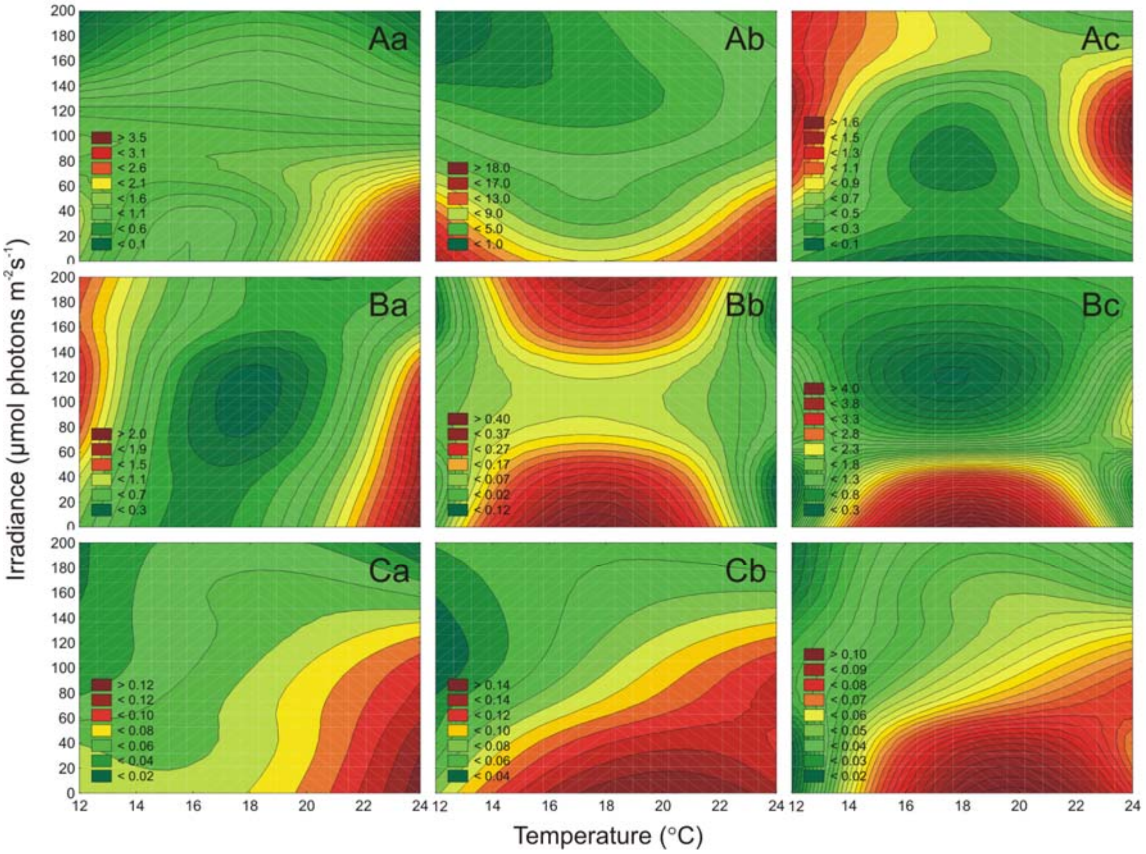

Figure S2. Changes in Phyco (A; pg·cell^{-1}), Chl *b* (B; pg·cell^{-1}), and Chl *c* (C; pg·cell^{-1}) content obtained after 7 days of experiment for airborne cyanobacteria: *Nostoc* sp. (Aa), *Synechococcus* sp. (Ab), and *Aphanothece* sp. (Ac); airborne green algae: *Oocystis* sp. (Ba), *Coccomyxa* sp. (Bb), and *Kirchneriella* sp. (Bc); airborne diatoms: *Nitzschia* sp. (Ca), *Amphora* sp. (Cb), and *Halamphora* sp. (Cc) under different irradiance (µmol photons m^{-2}s^{-1}) and temperature (°C) conditions.
